# Rapidly growing left atrial myxoma: a case report

**DOI:** 10.1186/1752-1947-5-417

**Published:** 2011-08-25

**Authors:** Ali Vazir, Harriet Douthwaite

**Affiliations:** 1 Royal Brompton Hospital, National Heart and Lung Institute, Imperial College London, Sydney Street, London SW3 6NP, UK; 2 The Whittington Hospital, Magdala Avenue, London N19 5NF, UK

## Abstract

**Introduction:**

Left atrial myxomas are rare benign tumors of the heart. They vary widely in size, and very little is known about their growth rate. The reported growth rates of left atrial myxomas from several published case reports appears to vary from no growth, to between 1.3 to 6.9 mm/month in diameter within patients with established myxoma who have not undergone surgery.

**Case presentation:**

We present the case of a rapidly growing pedunculated left atrial myxoma in a 62-year-old asymptomatic Caucasian woman found incidentally during routine transthoracic echocardiography. Our patient was attending her annual valve clinic assessment for moderate aortic regurgitation, and her two previous consecutive transthoracic echocardiography scans performed 12 and 24 months prior to this appointment had demonstrated a clear left atrium and aortic regurgitation of moderate severity.

**Conclusions:**

To the best of our knowledge, our case is the first to provide images of absence and presence of myxoma from transthoracic echocardiography scans taken a year apart, with estimated growth rate of 2.2 mm/month. Rapidly growing myxoma may be mistaken for thrombus, and may require urgent surgical excision to reduce the risk of associated complications such as thrombo-embolic events, sudden cardiac death and removal of a possibly malignant tumor. The potential for rapid growth should be considered if there is a plan to delay surgery. Furthermore, it would be pertinent to consider annual echocardiography in patients presenting with clinical features suggestive of cardiac myxoma such as constitutional symptoms, as these tumors may be rapid growing and may only become apparent on subsequent echocardiography.

## Introduction

Left atrial (LA) myxomas are rare benign tumors of the heart. They vary widely in size, and very little is known about their growth rate. The reported growth rates of LA myxomas from previous case reports vary from no growth, to between 1.3 to 6.9 mm/month in diameter of the myxoma [1,2]. These estimated growth rates are primarily assumed to be based on linear growth and have been calculated using original and follow up echocardiography images in case reports of patients with established LA myxomas, in patients who either refused or were unsuitable for cardiac surgery.

## Case presentation

**Figure 1:**
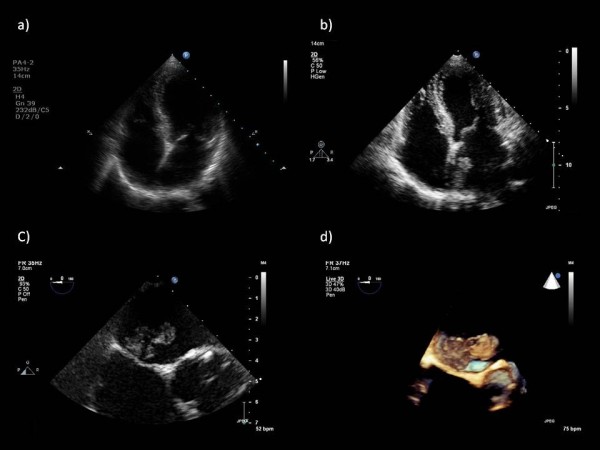
**Echocardiography**. (A) Transthoracic echocardiogram showing apical four-chamber view and clear left atrium performed a year prior to clinic attendance. (B) Transthoracic echocardiogram showing apical four-chamber view and incidental finding of left atrial myxoma at roof and interatrial septum. (C) Two-dimensional trans-esophageal echocardiogram (TEE) in mid-esophageal four-chamber view, modified and zoomed in on the interatrial septum demonstrating left atrial myxoma attached to the roof of the left atrium and the interatrial septum just above the left ventricular outflow tract and aortic valve. (D) Three-dimensional image of the left atrial myxoma taken from the same TEE mid-esophageal view as in (C). This figure clearly shows the broad peduncle of the myxoma and its attachment to the superior interatrial septum and roof of the left atrium. The three-dimensional TEE assisted the surgeons, by confirming the location of the left atrial myxoma and aiding the planned surgical route.

An asymptomatic 62-year-old Caucasian woman with moderate aortic regurgitation (AR) attended her third annual valve clinic. The AR was discovered incidentally after an examination by her medically-trained daughters. Her medical history was otherwise unremarkable. She had a heart rate of 80 beats/minute in sinus rhythm, blood pressure of 130/68 mmHg and normal carotid pulse, ejection systolic and early diastolic murmurs. A transthoracic echocardiogram (TTE) performed during the clinic visit showed evidence of good left ventricular systolic function and moderate AR. The TTE incidentally showed a large pedunculated left atrial (LA) mass measuring 26.7 × 10 mm at the roof of the LA, attached to the interatrial septum (Figure 1B). Her previous routine TTEs performed in the last two consecutive years demonstrated a clear LA (Figure 1A). Subsequently, two-dimensional and three-dimensional trans-esophageal echocardiography (TEE) confirmed a pedunculated LA mass, attached to the roof of the LA and the interatrial septum (Figure 1C, D). No other masses were found. Our patient underwent urgent cardiac surgery with complete excision of the LA mass. Histopathology results were consistent with myxoma. Our patient has remained asymptomatic for two years after surgery, with no recurrence of myxoma on TTE.

## Discussion

In the present report we describe a case of LA myxoma, with a rapid growth rate, found incidentally on routine TTE for assessment of AR. The latter was also discovered incidentally in the previous three years. Our case is unique as it is the first to present images of absence and presence of myxoma from TTE scans taken a year apart. Myxomas vary widely in size, and very little is known about their growth rates. In our case no mass was apparent on the TTE performed 12 months prior to surgery, implying a growth of 26.7 mm in the longest diameter of the myxoma over a 12-month period, equating to a minimum linear growth rate of 2.2 mm/month along the longest diameter of the myxoma. Previously published case reports have measured growth rates in patients with established LA myxoma with serial echocardiography performed, as patients would not be undergoing surgery for the reason described above [1,2].

## Conclusions

To the best of our knowledge, our case report is the first to present images of absence and presence of myxoma from TTE scans taken a year apart, with an estimated growth rate of 2.2 mm/month. The major differential diagnosis of myxoma is thrombus, which often has a rapid growth rate. Thus, caution is required in using rapid growth rate as a diagnostic criterion to differentiate between thrombus and myxoma. Myxomas may also resemble malignant tumors. A large atrial mass requires urgent surgical excision to reduce the risk of associated complications such as thrombo-embolic events, sudden cardiac death, and removal of possibly malignant tumor. The potential for rapid growth should be considered if there is a plan to delay surgery. Furthermore, it would be pertinent to consider annual echocardiography in patients presenting with clinical features suggestive of cardiac myxoma such as constitutional symptoms, as these tumors may be rapidly growing, and may only become apparent on subsequent echocardiography.

## Consent

Written informed consent was obtained from the patient for publication of this case report and any accompanying images. A copy of the written consent is available for review by the Editor-in-Chief of this journal.

## Competing interests

The authors declare that they have no competing interests.

## Authors' contributions

AV was responsible for obtaining our patient's history, her examination, for echocardiography data from our patient and writing the manuscript. HD was involved in writing and critical revision of the manuscript. All authors read and approved the final manuscript.
